# Progress of the National Insurance Bill

**Published:** 1911-08-12

**Authors:** 


### HOSPITALS AND STATE INSURANCE.

### PROGRESS OF THE NATIONAL INSURANCE BILL.

The National Insurance Bill, in its passage through the Committee of the House of Commons, has now reached a stage at which it is possible to form a good idea of the effect of the amendments that have been made. The clauses mainly affecting the relationship of hospitals, medical practitioners and dispensers to the proposed scheme have been dealt with, and it will not be altogether without profit to review briefly what has been done during the twelve days that have been spent in discussing the first seventeen clauses. The question of the composition of the various administrative bodies has yet to be considered, but no further progress will be made with the measure until the House meets for the autumn session at the end of October or the beginning of November, as the remainder of the present session, which will be brought to a close on August 18, will be fully occupied with other matters. The first important alteration in the Bill was made in Clause 1, the Chancellor accepting an amendment providing that no person with an income of over £160 a year should become a voluntary contributor. Thus, at the outset, the larger portion of the scheme relating to voluntary contributors was dropped. Another alteration of some importance was made in Clause 8, an amendment being agreed to, providing that a married mother who worked and was insured should receive sick pay of 7s. 6d. a week in addition to maternity benefit. In Clause 12, as reported in The Hospital last week, an amendment was made by which the sick pay of insured persons (with the exception of Post Office contributors) who have no dependents, and are inmates of hospitals, may be paid to such hospitals toward the cost of maintenance, and by a fundamental alteration of Clause 13, the administration of the medical benefit has been transferred from Friendly Societies to the Health Committees.

The difference between Clause 14 as amended and as originally drafted is so great that it is difficult to recognise the relationship between the two, the new clause being something like four times as long as the old cluase. With the exception that the demand for a reduction of the income limit of compulsorily insured persons has not been satisfied, practically all the claims made by medical practitioners have been satisfied. There has even been a sort of compromise in regard to the income limit, for although the amendment, moved by Sir Philip Magnus, providing that insured persons earning over £2 a week should receive the benefit and make their own medical arrangements, was negatived, a related amendment moved by Dr. Addison was adopted by 279 to 41 votes. Dr. Addison's amendment empowers local Health Committees to require persons whose incomes are above a limit to be fixed by them to make their own arrangements for medical attendance, and to permit persons whose incomes are below such a limit to make their own arrangements if they desire to do so, the Health Committees contributing a calculated sum towards the payment of medical fees, and the amendment safeguards the interests of "contract" institutions. The clause now provides that the Health Committees shall make arrangements with duly qualified medical practitioners in accordance with regulations to be made by the Insurance Commissioners; these regulations are to require the adoption by every local Health Committee of such a system as will practically reserve a free choice of medical attendant by insured patients. As to the method of remuneration, this will be left to the Health Committees, and in all probability different methods will be adopted in different districts. If in any year the amount payable to a local Health Committee in respect of all persons for the administration of whose medical benefit it is responsible is insufficient to meet the estimated expenditure thereon, the deficit may be made up by the Treasury and County Councils, so that medical practitioners are likely to receive more adequate remuneration than would be the case if the medical benefit were administered by Friendly Societies with definite sums set aside for this purpose. A number of amendments have also been made affecting the dispensing of medicines and the supply of drugs, and the difficulties explained in The Hospital of July 22nd (page 421) have to a large extent been overcome. Arrangements for the supply of drugs are to be made with legally qualified persons, and remuneration is to be made according to official schedules of prices to be fixed locally; the original intention of separating the functions of prescribers and dispensers has been definitely embodied in the clause.

It was expected when Clause 15, which makes provision for the administration of sanatorium benefit, was reached that the question of assistance to hospitals would be raised, but Mr. McKenna stated that this could be better discussed on Clause 47. In Clause 16, which deals with the administration of maternity benefit, an amendment was made providing that a mother should have the right to decide whether she would be attended by a registered medical practitioner or by a duly certified midwife, and should have free choice in the selection of such practitioner or midwife. Clause 17, which empowers approved societies to grant subscriptions or donations to hospitals, etc., was amended so as to provide that nurses might be appointed for the purpose of visiting insured persons, whether members of a society or not. These clauses will, of course, be subject to revision at the report stage.

